# Kidney Biopsy in a Pregnant Patient with Suspected Glomerular Disease: PRO

**DOI:** 10.34067/KID.0000000000000242

**Published:** 2023-10-26

**Authors:** Andrea L. Oliverio, Elizabeth M. Hendren

**Affiliations:** 1 Department of Internal Medicine, Division of Nephrology, University of Michigan, Ann Arbor, Michigan; 2 Department of Medicine, Division of Nephrology, University of British Columbia, Vancouver, British Columbia, Canada

**Keywords:** glomerular disease, GN, glomerulopathy, kidney biopsy, renal biopsy, women's health

## Introduction

Kidney biopsies have long been avoided in pregnancy because of concerns of maternal and fetal complications. In this debate, we argue that a kidney biopsy in a patient with suspected glomerular disease can provide essential information that justifies the potential risk, and we advocate that counseling about a kidney biopsy in pregnancy be a shared, patient-centered discussion without distortion of these potential risks.

CKD is rare among reproductive aged patients and estimated to affect about 3% of pregnancies.^1^ However, among those presenting with proteinuric diseases of pregnancy (gestational proteinuria and preeclampsia), the rate of undiagnosed CKD is likely higher.^2^ In addition, pregnancy is often a time of regular health investigations which can reveal impaired kidney function or proteinuria, and pregnancy itself may serve as an unmasking or triggering event bringing glomerular disease to attention. Notably, while pregnancy induces several physiologic changes in the kidney, including vasodilation and hyperfiltration, an increase in proteinuria is modest, typically <300 mg/24 hours.^3^ Beyond this, particularly when proteinuria develops before 20 of weeks gestation, a glomerular disease should be on the differential.

## Benefits of Kidney Biopsy

Kidney biopsy is the gold standard diagnostic test for glomerular disease and should be undertaken when the results are expected to alter treatment.^4^ Histopathologic evaluation of kidney tissue elucidates or confirms a diagnosis; may reveal unexpected or additional glomerular, tubular, or interstitial pathology; and provides information regarding degree of activity and chronicity. This information can have significant therapeutic implications.

When patients are carefully selected, kidney biopsies performed during pregnancy can be high yield, informing both kidney and pregnancy management. Performing a biopsy for new-onset proteinuria or AKI in a young patient frequently leads to a diagnosis of a *de novo* glomerular disease including lupus nephritis, FSGS, or IgA nephropathy. A biopsy is essential for differentiating among the potential etiologies because the two most common glomerular diseases in pregnancy, FSGS and IgA nephropathy, have no serologic markers. Genetic conditions which may arise in the childbearing age group, including Alport syndrome and Fabry disease, can be quickly diagnosed with a kidney biopsy, wherein genetic testing may take weeks or be more expensive or inaccessible to patients. Furthermore, the appropriate treatment of lupus nephritis is typically guided by pathologic features, including World Health Organization Class and activity and chronicity scores. Therefore, a biopsy is essential for the accurate and timely diagnosis and treatment of patients with suspected glomerular disease.

Glomerular diseases can be treated with pregnancy-safe medications such as prednisone and calcineurin inhibitors, resulting in improved kidney function and proteinuria and decreasing risk for adverse pregnancy outcomes. A systematic narrative review of kidney biopsies performed in pregnancy demonstrated that 39 of 59 biopsies (66%) resulted in therapeutic change, specifically the addition of immunosuppressive medications or the prolongation of pregnancy.^4^ In a recent case series of 20 pregnant patients who underwent kidney biopsy between 11 and 30 weeks for significant proteinuria, biopsy-confirmed diagnosis informed treatment: 17 of 20 patients received immunosuppression and had improvement in proteinuria, and three patients avoided immunosuppression because they were found to have unexpected advanced glomerulosclerosis or diabetic nephropathy.^5^ A case series of biopsies performed in 11 pregnant patients with presumptive lupus flares identified proliferative lupus nephritis in 91% of patients, and the authors thoughtfully describe how five patients weighed this information with their values to determine whether to continue or terminate their pregnancy and how this affected their subsequent medical treatment as well.^6^

A biopsy can also elucidate the etiology and determine treatment in kidney transplant patients with new-onset graft dysfunction and is recommended when necessary.^7,8^ A case report of two transplant kidney biopsies in pregnancy, performed at 20 and 23 weeks' gestation, juxtaposed how meaningfully excluding or confirming preeclampsia when it could not otherwise be determined assisted in the prolongation of one pregnancy and the delivery of another.^9^ Although soluble fms-like tyrosine kinase-1 and placental growth factor (PlGF) testing in pregnancy has showed promising results to help rule in or rule out preeclampsia,^10,11^ this test is only very recently approved in the United States and not yet widely available, nor validated in people with kidney disease. With an established diagnosis other than preeclampsia, pregnancy-safe treatments can potentially be pursued, proteinuria and kidney function monitored closely, and gestation prolonged, lessening the likelihood of fetal complications of prematurity.

## Biopsy Safety and Risks

A percutaneous kidney biopsy is typically performed with an automated device under imaging, most commonly ultrasonography, and in the prone position. A recent meta-analysis estimated complication rates from more than 110,000 biopsies and reported macroscopic hematuria in 3.5%, perinephric hematoma in 11%, and bleeding requiring blood transfusion in 1.5%.^12^ In pregnancy, performing a kidney biopsy becomes more technically challenging with growth of a gravid uterus and is often completed in the lateral decubitus position in later stages of pregnancy. In addition, higher kidney blood flow in pregnancy can increase the likelihood of bleeding. However, in a systematic narrative review of 11 studies including 197 antepartum biopsies and their complications from 1982 to 2012, minor complications such as macrohematuria and hematomas not requiring transfusion occurred in only 5% of cases.^5^ Four major complications were reported (2%), all of which included major bleeding with perinephric hematomas requiring blood transfusion. Immediate fetal complications associated with biopsy were rare: One twin gestation developed placental abruption and preterm delivery, and one singleton gestation had preterm birth with perinatal death. There were no maternal deaths. These major complications occurred at a median of 25 weeks' gestation (range 23–26 weeks).^4^ While major complication rates of kidney biopsy in pregnancy are similar to that of all-comers, total complication rates are significantly higher than biopsies performed after pregnancy (7% versus 1%) likely because of this otherwise healthier patient population.^4^

Since Piccoli's systematic review, another case series of 11 pregnant people with known lupus biopsied at a median of 16 weeks (9–27 weeks) reported no known complications resulting from biopsy.^6^ Finally, the case series of 20 patients in Mexico reported 1 of 20 (5%) experienced major bleeding and hematoma requiring blood transfusion; this biopsy was performed at 11 weeks' gestation.^5^ Of note, in this series, most patients had kidney biopsies performed between 12 and 26 weeks' gestation, with only four patients biopsied after 26 weeks. In all cases described, biopsies were diagnostic with adequate tissue for analysis.^5,6^ Transplant patients were excluded from Piccoli's systematic review, and while reports of transplant kidney biopsy in pregnancy are few, it is believed to be better tolerated because of graft location.^9^

In summary, while risks of complications of a kidney biopsy are higher in pregnancy than postpartum, they are similar to that of all patients who undergo a kidney biopsy. Therapeutic changes are common after biopsy, and the data obtained can be pivotal in informing decisions that affect both maternal and fetal health. The most severe complications have been described late in the second trimester at 23–26 weeks' gestational age. Although more recent series described that biopsies have safely been performed at up to 30 weeks' gestation, the data are still very limited on biopsies performed after 26 weeks. The benefit of obtaining biopsy is likely greatest earlier in pregnancy, when the risks of major complications are lower, and there is greater advantage in precisely defining the diagnosis and prognostic features to inform management.

## Engaging in Shared Decision Making about Kidney Biopsy in Pregnancy

Ultimately, the decision to undergo a kidney biopsy in pregnancy is one a patient must make fully informed by one's nephrologist and high-risk obstetrician. Individual risk tolerance, comfort with uncertainty, and values and priorities related to treatment attributes will inform patients' decision making. Individual and local resources may also influence decision making. In some settings, serologic and genetic testing may be unavailable, costly, and/or untimely, and a kidney biopsy can be the most parsimonious and effective way to obtain data that informs management both in pregnancy and thereafter. In the United States, pregnant women are eligible for Medicaid coverage which extends 60 days after birth; coverage beyond this has been expanded in some but not all states. Delaying biopsy until postpartum may have different challenges in access to appropriate outpatient care particularly for patients with maternal morbidity at delivery.^13^ Finally, there may be local factors relevant to decision making, including the ability to manage biopsy complications, access higher levels of needed neonatal care exemplified in a recent case report,^14^ or access therapeutic termination. All these factors may be considered when weighing a potential kidney biopsy.

In addition, we must acknowledge our own cognitive and societal biases when considering risks in pregnancy. A Hastings Center Report thoughtfully pointed out that in pregnancy, medicine is often oriented toward the risks of interventions (in this case, biopsy) with less consideration of the risks of failing to intervene. In society, the ideal of a self-sacrificing good mother can lead to the judgment that any fetal risk can trump considerations important to the woman herself, including examples from diet to antidepressants.^15^ While the risk/benefit ratio needs to be examined carefully in pregnant women, there are many situations where biopsy is withheld because of fear of complications that are not definitively supported by evidence and are similar to the general population pursuing a kidney biopsy. This should be shared with patients, so they can make an informed decision.

Ultimately, while a kidney biopsy in pregnancy may carry higher risk than a native *postpartum* kidney biopsy, it can generally be performed safely with a <10% risk of complications, largely minor. In thoughtfully selected patients, a kidney biopsy will likely provide diagnostic information and guide therapy. This will facilitate the ability for patients to make autonomous, informed decisions and can optimize the health of the pregnant person, as well as the developing fetus.
